# Supplementary material for: Fecal Metabolomics Reveals the Foraging Strategies of Giant Pandas for Different Parts of Bamboo
Source: Animals (Basel). 2023 Apr 7;13(8):1278. doi: 10.3390/ani13081278 (PMC10135075; doi:10.3390/ani13081278)
Supplement: Supplementary file 1 [file animals-13-01278-s001.zip › supporting information Figure S1-S4.pdf]

Supporting figures

Figure S1&S2. Total ion current (TIC) overlap map of positive and negative ion modes of quality control (QC) samples

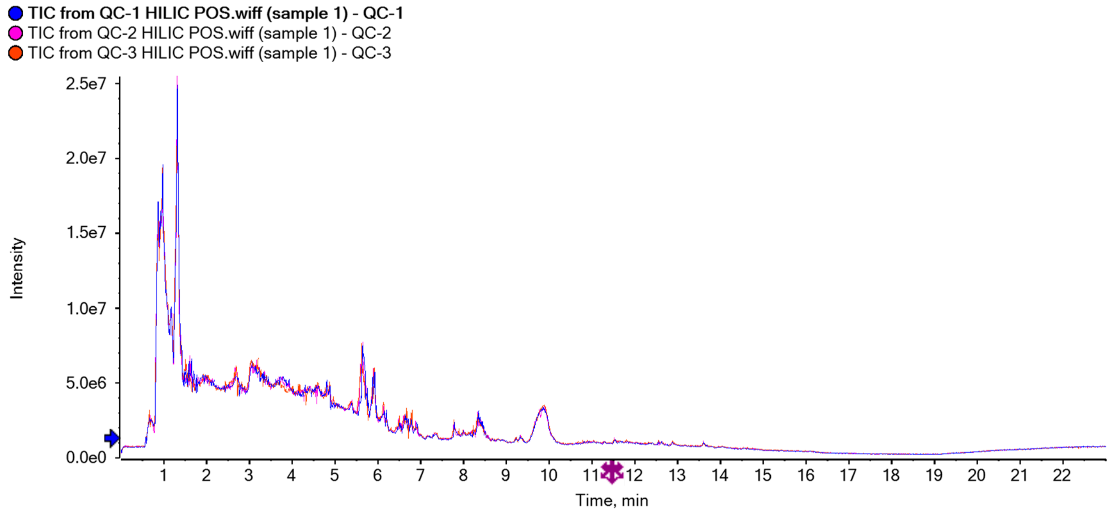

Figure S1 TIC overlap map of positive ion modes of QC samples

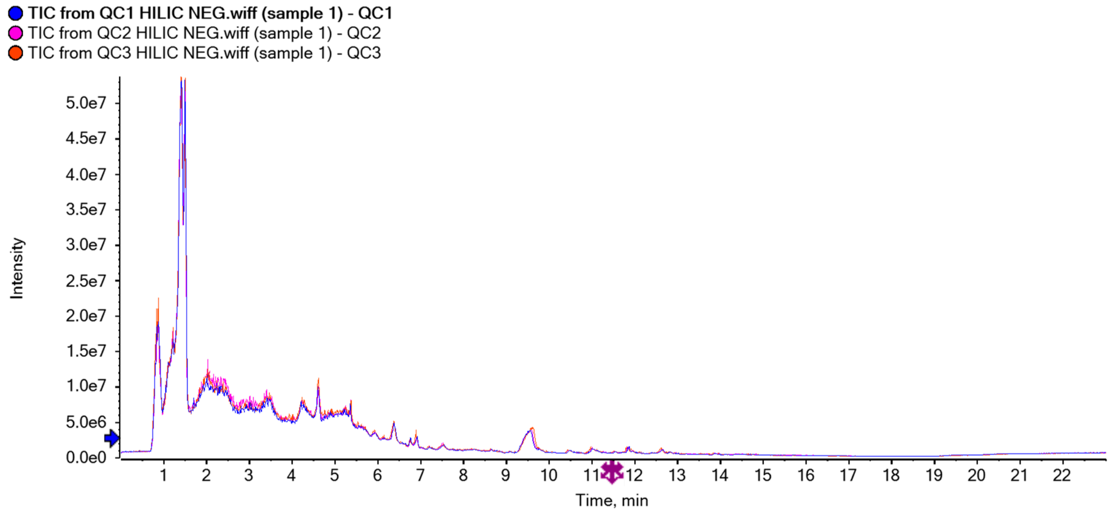

Figure S2 TIC overlap map of negative ion modes of QC samples

Figure S3 & S4. Orthogonal partial least squares discriminant analysis (OPLS-DA) of fecal metabolites in giant pandas under different dietary conditions

In order to obtain a higher level of group separation and a better understanding of the variables responsible for group classification, OPLS-DA was applied. Seven-fold

cross validation was used to estimate the optimal component number of the model and avoid the over-fitting. At positive ion mode, the R^2Y and Q^2accumulation were R^2Y= 0.999, Q^2 =0.984 (Culm vs Shoot); R^2Y= 0.996, Q^2 =0.981 (Leaf vs Shoot); R^2Y= 0.989, Q^2 =0.963 (Leaf vs Culm). At negative ion mode, the R2 and Q2 intercept values were R^2Y= 0.998, Q^2 = 0.986 (Culm vs Shoot); R^2Y= 0.994, Q^2 = 0.982 (Leaf vs Shoot); R^2Y= 0.986, Q^2 = 0.965 (Leaf vs Culm). The values of R^2Y and Q^2 accumulation were close to 1, indicate the models were stable and reliable. The R^2 and Q^2 intercept values after 200 permutations were shown in figure. The low values of the Q^2 intercept indicate the robustness of the models, and thus show a low risk of over fitting and reliability.

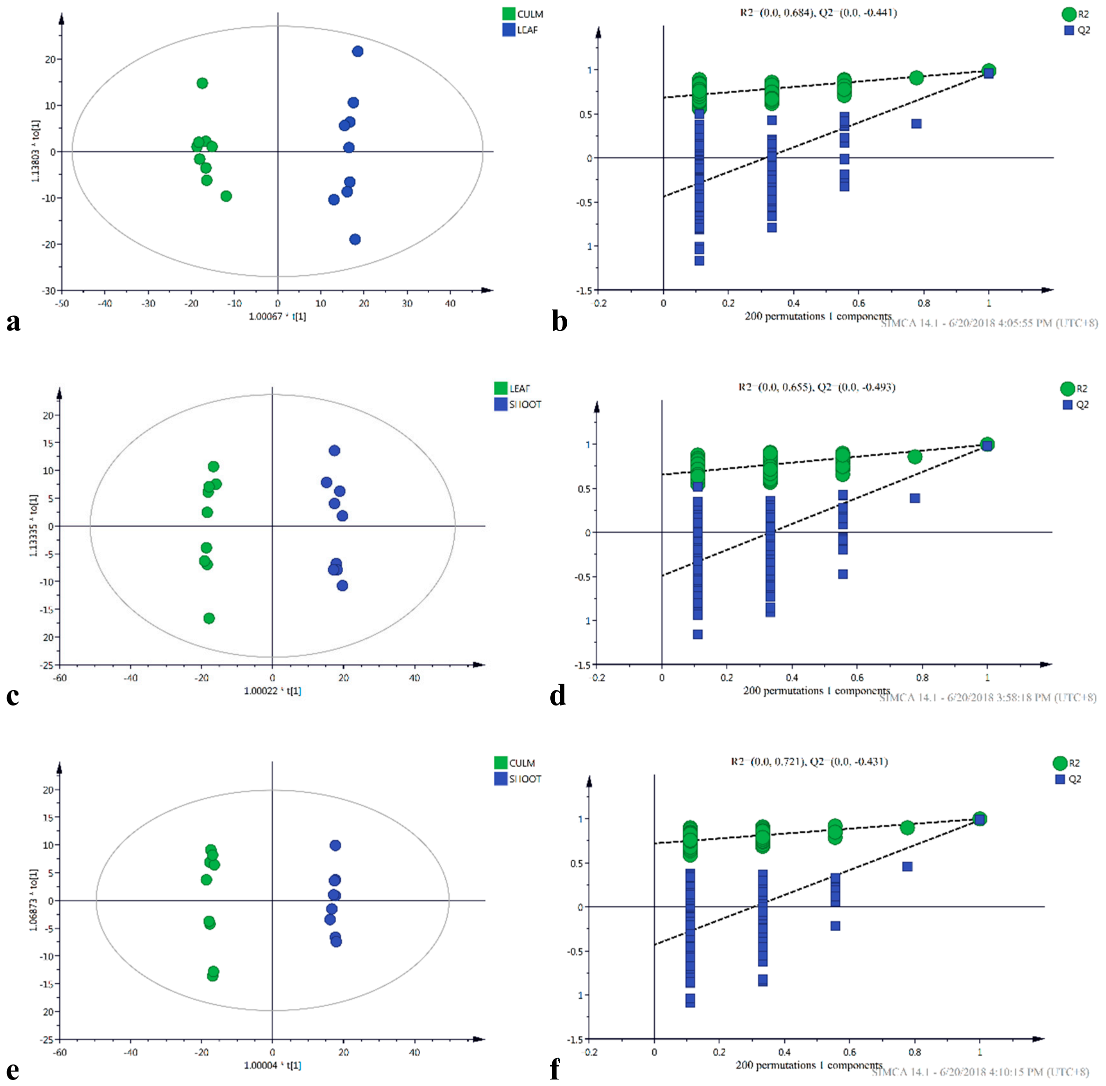

Figure S3 Positive ion pattern OPLS-DA score diagram and permutations test.

Score plot of OPLS model obtained from Culm vs Leaf (a); Leaf vs Shoot (c); Culm vs Shoot (e). Permutation analysis of OPLS-DA models from Culm vs Leaf (b); Leaf vs Shoot (d); Culm vs Shoot (f). Two hundred permutations were performed, and the resulting R2 and Q2 values were plotted. (Green triangle): R2; (blue square): Q2. The green line represents the regression line for R2 and the blue line for Q2.

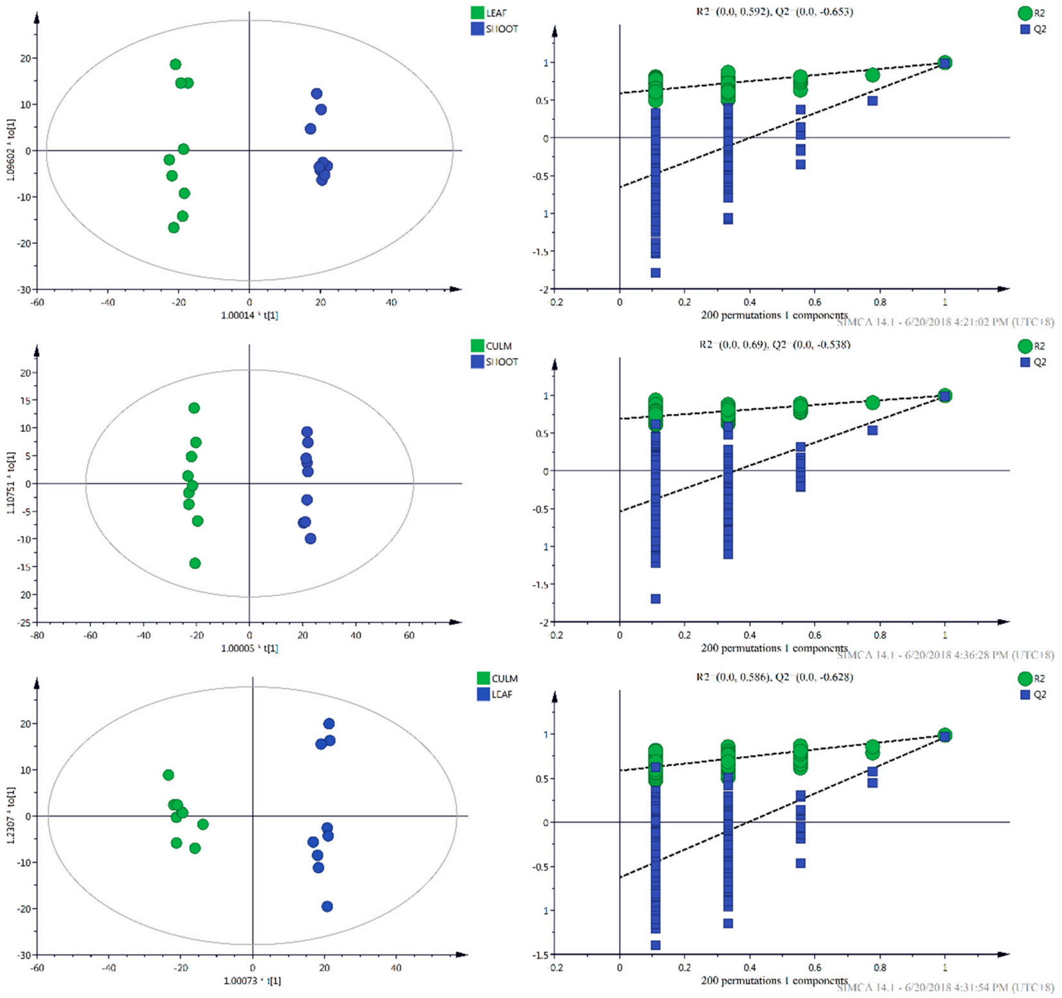

Figure S4 Negative ion pattern OPLS-DA score diagram and permutations test. Details were described in figure s3.
